# 532 The Brisbane Burn Scar Impact Profile: Score Changes over Time in Adult Patients

**DOI:** 10.1093/jbcr/irae036.166

**Published:** 2024-04-17

**Authors:** Martin R Buta, Sarah Findeisen, Pengsheng Ni, Lewis E Kazis, Sean Hickey, Jonathan S Friedstat, John T Schulz, Colleen M Ryan, Jeffrey C Schneider, Jeremy Goverman

**Affiliations:** Massachusetts General Hospital, Boston, MA; Boston University School of Public Health, Boston, MA; Department of Health Law, Policy and Management, Boston University School of Public Health, Boston, MA; Massachusetts General Hospital/Shriners Children's, Boston, MA; Spaulding Rehabilitation Hospital/Harvard Medical School, Boston, MA; Massachusetts General Hospital, Boston, MA; Boston University School of Public Health, Boston, MA; Department of Health Law, Policy and Management, Boston University School of Public Health, Boston, MA; Massachusetts General Hospital/Shriners Children's, Boston, MA; Spaulding Rehabilitation Hospital/Harvard Medical School, Boston, MA; Massachusetts General Hospital, Boston, MA; Boston University School of Public Health, Boston, MA; Department of Health Law, Policy and Management, Boston University School of Public Health, Boston, MA; Massachusetts General Hospital/Shriners Children's, Boston, MA; Spaulding Rehabilitation Hospital/Harvard Medical School, Boston, MA; Massachusetts General Hospital, Boston, MA; Boston University School of Public Health, Boston, MA; Department of Health Law, Policy and Management, Boston University School of Public Health, Boston, MA; Massachusetts General Hospital/Shriners Children's, Boston, MA; Spaulding Rehabilitation Hospital/Harvard Medical School, Boston, MA; Massachusetts General Hospital, Boston, MA; Boston University School of Public Health, Boston, MA; Department of Health Law, Policy and Management, Boston University School of Public Health, Boston, MA; Massachusetts General Hospital/Shriners Children's, Boston, MA; Spaulding Rehabilitation Hospital/Harvard Medical School, Boston, MA; Massachusetts General Hospital, Boston, MA; Boston University School of Public Health, Boston, MA; Department of Health Law, Policy and Management, Boston University School of Public Health, Boston, MA; Massachusetts General Hospital/Shriners Children's, Boston, MA; Spaulding Rehabilitation Hospital/Harvard Medical School, Boston, MA; Massachusetts General Hospital, Boston, MA; Boston University School of Public Health, Boston, MA; Department of Health Law, Policy and Management, Boston University School of Public Health, Boston, MA; Massachusetts General Hospital/Shriners Children's, Boston, MA; Spaulding Rehabilitation Hospital/Harvard Medical School, Boston, MA; Massachusetts General Hospital, Boston, MA; Boston University School of Public Health, Boston, MA; Department of Health Law, Policy and Management, Boston University School of Public Health, Boston, MA; Massachusetts General Hospital/Shriners Children's, Boston, MA; Spaulding Rehabilitation Hospital/Harvard Medical School, Boston, MA; Massachusetts General Hospital, Boston, MA; Boston University School of Public Health, Boston, MA; Department of Health Law, Policy and Management, Boston University School of Public Health, Boston, MA; Massachusetts General Hospital/Shriners Children's, Boston, MA; Spaulding Rehabilitation Hospital/Harvard Medical School, Boston, MA; Massachusetts General Hospital, Boston, MA; Boston University School of Public Health, Boston, MA; Department of Health Law, Policy and Management, Boston University School of Public Health, Boston, MA; Massachusetts General Hospital/Shriners Children's, Boston, MA; Spaulding Rehabilitation Hospital/Harvard Medical School, Boston, MA

## Abstract

**Introduction:**

Hypertrophic scars can result from burn injury, often leading to a long-term recovery that involves surgical reconstruction and rehabilitation. The Brisbane Burn Scar Impact Profile (BBSIP) is a patient-reported health-related quality of life survey designed to assess the impact of burn scars in children and adults. In this study, we aimed to assess the longitudinal outcomes of changes of BBSIP scores in adult patients with hypertrophic burn scars undergoing reconstruction and/or laser treatment.

**Methods:**

A prospective study enrolled 113 adult patients with hypertrophic burn scars undergoing reconstruction, including skin grafts, and/or laser treatment at a major North American tertiary care center. Patient demographics, percent total body surface area (TBSA) burned, and burn etiology were obtained. A total of 238 surveys were collected after a reconstructive or laser treatment. Using exploratory and confirmatory factor analysis (FA) on the 7 sub-domains of the BBSIP, we developed an overall impact scale from two subscales: factor 1 and factor 2 were each comprised of 5 items. BBSIP scores were calculated as the average item scores within each scale and standardized to a t-score using scores from respondents who first completed the survey. We assessed the impact of demographics on BBSIP scores over time using a linear mixed model. We used a BIC (Bayesian information criteria) value to determine the optimal model by adding the interactions between time since injury and the independent demographic variables. We calculated regression coefficients (95% confidence interval) and determined statistical significance at p< 0.05.

**Results:**

Of the 113 patients (59 female, 54 male), 61 (54%) completed the survey only once, 20 (17.7%) completed 2 surveys, and 16 (14.2%) completed 3 surveys. For the factor 2 scores, the three sub-domains (relationships and social interactions, appearance, and emotion) were found to decrease over time since burn injury, with males having about 0.7 standard deviation lower scores than females. Males were found to have lower scores than females for all sub-domains except for factor 1’s work and daily activities scores. Skin grafts resulted in decreasing scores for itch, pain, and other sensations with longer periods of time from burn injury. Skin grafts also resulted in decreasing scores for work and daily activities with increasing time from burn injury for subjects with TBSA>5%.

**Conclusions:**

This study demonstrates how BBSIP scores may change over time in adult patients with hypertrophic burn scars undergoing reconstruction and/or laser treatment. A deeper understanding of the anticipated trajectory of quality of life measures in these patients can help guide burn care providers in developing long-term management plans.

**Applicability of Research to Practice:**

This study helps clinicians understand how key health-related quality of life measures may evolve over time in adult burn patients with hypertrophic scars.

